# Editorial: Immunomodulatory role of metalloproteases in chronic inflammatory diseases

**DOI:** 10.3389/fimmu.2023.1196791

**Published:** 2023-04-11

**Authors:** Guan-Jun Yang, Dan Li, Chung Nga Ko, Shicheng Guo, Chao Yang

**Affiliations:** ^1^ State Key Laboratory for Managing Biotic and Chemical Threats to the Quality and Safety of Agro-products, Ningbo University, Ningbo, Zhejiang, China; ^2^ Laboratory of Biochemistry and Molecular Biology, School of Marine Sciences, Ningbo University, Ningbo, China; ^3^ State Key Laboratory of Southwestern Chinese Medicine Resources, School of Pharmacy, Chengdu University of Traditional Chinese Medicine, Chengdu, China; ^4^ C−MER Dennis Lam and Partners Eye Center, Hong Kong International Eye Care Group, Hong Kong, Hong Kong SAR, China; ^5^ Department of Medical Genetics, School of Medicine and Public Health, University of Wisconsin-Madison, Madison, WI, United States; ^6^ National Engineering Research Center for Marine Aquaculture, Institute of Innovation & Application, Zhejiang Ocean University, Zhoushan, China

**Keywords:** metalloproteases, immunomodulation, chronic inflammatory diseases, MMPs, ADAMs

Metalloproteases are a diverse class of enzymes involved in the regulation of numerous pathological and physiological processes. Evidence has shown that metalloproteases could either directly or indirectly regulate the secretion of chemokines and the differentiation and/or activation of immune cells, thereby mediating many inflammatory and innate immune responses (1, 2). While different metalloproteases could have substantially different primary structure, their active center which contains metal ions (e.g. iron, zinc, cobalt, nickel ions) is relatively conservative. Metalloprotease relies on metal ions to maintain its catalytic function. Studies have shown that metal chelators such as EDTA could completely inactivate metalloproteases (3–6; Liu et al.). Under inflammatory conditions, metalloproteases are constitutively activated or deactivated in multiple immune- or non-immune cells and could contribute to a variety of inflammatory diseases, such as rheumatoid arthritis (RA) (Li et al.), chronic enteritis (Deng et al. and Mei et al.), allergic dieseases (Wang and Wang and Bendavid et al.), diabetes (Chen et al.), and cancers (He et al.), etc. Mutiple chronic inflammatory diseases could even hijack various metalloproteases to promote and exacerbate inflammation (1, 2). Numerous preclinical and clinical studies have shown that metalloprotease modulators, including lysine-specific demethyalses (KDMs) inhibitors (3–5, 7), histone deacetylases (HDACs) inhibitors (8), and matrix metalloproteinases (MMPs) inhibitors (6), and a disintegrin and metalloproteinases (ADAMs) inhibitors (9), possess *in vitro* and *in vivo* anti–inflammatory activities. Therefore, understanding the roles of metalloproteinases in the immune system may potenitally uncover new targets for the diagnosis and treatment of chronic inflammatory diseases.

This Research Topic contributes to a better understanding of immunomodulatory role of the metalloproteases in several chronic inflammatory diseases and highlights the clinical significance of the immunomodulatory role of metalloproteases in disease diagnosis and drug discovery. This Research Topic accepted a total of 12 articles from 75 authors. All contributions to this Research Topic focus on one or more of the following research areas:

## MMPs

MMPs are a family of zinc-dependent proteases playing the role of targeting and cleaving extracellular proteins. They are involved in the occurence and progression of multiple chronic inflammatory diseases, including colitis [Chen et al. and Deng et al.], rheumatoid arthritis (RA) (Li et al.), diabetes (Chen et al.), and cancers (10), etc. Mei et al. identifed five MMPs-realted genes (TLR5, CD160, MMP-9, PTGDS, and SLC26A8) as the biomakers of inflammatory bowel disease (IBD) using machine learning by screening from public Gene Expression Omnibus datasets and functional enrichment analysis. *In vivo* study using sodium dextran sulfate (DSS)-induced colitis indicated that the level of TLR5 was significantly reduced in the model group and the levels of other four protiens were significantly increased. Futher studies have shown that MMPs modualate intestinally inflammatary and immune responses mainly through CD8+ cells in colitis. This study reveals the crucial roles of MMPs in the pathogenesis of IBD and provides insights into the molecualr mechanism and theranostical targets of IBD. Deng et al. suggests that MMPs-related modules are the main differential gene sets between Crohn’s disease and ulcerative colitis based on integrated analysis of multiple microarray. RA is an autoimmune disease caused by a variety of factors (Yang et al.). MMPs were found to play a crucial role in the pathogenesis of RA. Mutiple herbal medicines can inhibit the inflammatory responses of RA and thus alleviate RA through modulating MMPs and the associated signaling pathways (Li et al.). Diabetic ulcer is a serious complication of diabetes characterized by recalcitrant wounds, which could tremendously affect the quality of life of patients and impose a substantail medical and economic burden on a country. Chen et al. found that many natural products including flavonoids, alkaloids, polysaccharides, and polypeptides, etc. are effective to treat diabetic ulcer through regulation of the MMPs-mediated pathways. Furthermore, MMPs also contribute to many cancers. He et al. found that MMPs mediate the progression of colitis-associated cancer by regulating the expression of each member of MMPs precisely and thus promoting cell proliferation and differentiation, angiogenesis, and extracellular matrix remodeling. Wang et al. also suggested that MMPs could be theranostic targets of cancers and could potentially be applied in cancer diagnosis and treatment.

## ADAMs

ADAMs are a family of transmembrane and secreted metalloproteases. They are involved in many chronic inflammatory diseases through modulating proteolysis and the related signalling pathways (11). Wang et al. sumarized the structure and immunoregulatory roles of ADMAD17 in tumorigenesis and highlighted that aborgating ADMAD17 using small inhibitors or monoclonal antibodies is an effective strategy to combat cancers. In addition, Wang et al. also showed that ADAMs modulate the adhesion and migration of cancer cells *via* releasing the proteolytic cell surface molecules including adhesion molecules, growth factors, and precursor forms of cytokines. Devel et al. suggested that both MMPs and ADAMs are involved in regulating CD95/CD95L signaling in proteolytic enzyme-dependent manner and targeting this signalling is a potential strategy for fighting cancer. Bendavid et al. explored the role of ADMAD28 in asthma using an OVA-induced asthma model. This study found that ADMAD28 could increase collagen deposition, smooth muscle hyperplasia, mucous hyperplasia, suggesting that ADMAD28 could promote the progression of asthma through regulating airway remodeling.

## Others

Some enzymes such as Jumonji C (JmjC) demethylases, ten-eleven-translocation (TET) enzymes, COX2, and HDACs are also metalloproteases and are invlvoled in modulating several chronic inflammatory diseases [(5, 7), Jin et al. and Wang et al.]. Our previous studies showed that KDM5A or LSD1 could inhibit the progression of triple-negative breast cancer or acute leukemia *via* inducing cell cycle arrest and senescence, leading to cell apostosis *in vitro* and *in vivo* (12–15). Jin et al. systematically sumarized the emerging role of TET enzymes in the immune microenvironment at the maternal-fetal interface during decidualization and early pregnancy, providing an insight into the future implications in disease diagnosis or treatment. Zhou et al. revealed that the aqueous extract of *Dendrobium officinale* flowers exhibit anti-glycation, anti-cyclooxygenase, and anti-skin aging activity. HDAC1, which is a type of class I HDACs, is a crucial enzyme modulating the progression of chronic inflammatory diseases, including allergic diseases (16–19). Wang and Wang sumarized the roles of HDAC1 in allergic diseases. After stimulated by allergen, HDAC1 upregulates the levels of T helper 2 cytokine, reduces the number of Th1/Th17 cells and Interleukin-10, and downregulate the expression of TWIK-related potassium channel-1. This review hightlights the functions and regulatory roles of HDAC1 in allergic diseases, aids the understanding of allergic multimorbidity relationships, as well as provides insight into the feasibility of using HDAC1 as a molecular target for the diagnosis and treatment of allergic diseases.

In summary, we anticipate that this Research Topic will inspire future research on the immunoregulatory roles of metalloproteinases in chronic inflammatory diseases. Understanding the function and regulatory mechanisms of metalloproteinases may provide insights into the future development of diagnostic and therapeutic approaches (e.g. nanomaterials or metal-based probe targeting these enzymes) for chronic inflammatory diseases (20–24).

## Author contributions

All authors listed have made a substantial, direct, and intellectual contribution to the work and approved it for publication.
